# Smart Indicator for Fish Freshness Monitoring: A Chitosan/Gelatin/Zinc Oxide Nanoparticle Composite Incorporating Pomegranate Flower Anthocyanin Extract Nanocapsules

**DOI:** 10.1002/fsn3.70944

**Published:** 2025-09-22

**Authors:** Solmaz Choubaki, Homa Baghaei, Abdorreza Mohammadi Nafchi

**Affiliations:** ^1^ Department of Food Science & Technology, Da.C. Islamic Azad University Damghan Iran; ^2^ Food Technology Division, School of Industrial Technology Universiti Sains Malaysia Minden Penang Malaysia

**Keywords:** anthocyanins, biodegradable film, fish spoilage indicator, pH‐responsive packaging, pomegranate flower extract

## Abstract

This study aimed to develop a smart film for monitoring pH changes, utilizing chitosan/gelatin/nano zinc oxide (ZnO) enhanced with nanocapsules of pomegranate flower extract (PFEN). PFEN were prepared via spray drying with a maltodextrin‐Arabic gum coating. The resulting chitosan/gelatin films were modified with nano ZnO (1%) and varying PFEN concentrations (1%, 2%, and 3%). The physicochemical, mechanical, optical, barrier, structural, crystallinity, thermal stability, antioxidant, and antibacterial properties of the films were comprehensively evaluated. The colorimetric response of the smart films to pH changes, anthocyanin release, and the film's potential for indicating fish freshness were also assessed. Incorporation of PFEN significantly enhanced water vapor barrier (from 6.37 to 2.94 × 10^−11^ g m/m^2^ day), tensile strength (by ~20%), and antioxidant activity (DPPH scavenging up to 78.9%) (*p* < 0.05). FTIR spectroscopy confirmed the compatibility of polymers and additives. SEM imaging revealed that the addition of PFEN increased film surface roughness. Films containing PFEN exhibited a clear color response to varying pH levels. The films exhibited distinct pH‐dependent color changes (Δ*E*), correlating with increasing TVB‐N values in trout fillets stored at 4°C, indicating effective freshness monitoring. The enhanced mechanical, antioxidant, and antimicrobial properties suggest that these films are suitable for use as active packaging to extend the shelf life of meat products.

## Graphical abstract

This study developed smart chitosan/gelatin films incorporating nano ZnO and pomegranate flower extract nanocapsules (PFEN) to monitor pH changes and indicate fish freshness. The films exhibited enhanced mechanical, antioxidant, and antimicrobial properties, as well as a clear colorimetric response from red to green correlating with spoilage, making them promising for active food packaging.

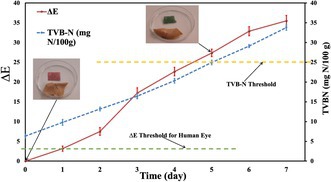

## 1 Introduction

Fishery products are among the most widely consumed food products in the world; however, these products are perishable and have a limited shelf life in the refrigerator (Tavakoli et al. 2023). During the spoilage of food products rich in protein, such as various types of meat, volatile nitrogen compounds are produced due to the growth and activity of bacteria and internal enzymes, which alter the pH of the product (Dong et al. 2023). Recognizing the freshness of these perishable products is crucial, which has driven growing interest in developing intelligent detector films sensitive to pH changes in recent years (Zhang et al. 2023). These intelligent films comprise two main components: a polymer substrate and a pH‐sensitive pigment component (Khajeh et al. 2024; Samani et al. 2023).

Chitosan is a natural cationic polysaccharide with a high molecular weight obtained from the deacetylation of chitin. Chitosan is biodegradable, biocompatible, and non‐toxic, with good antimicrobial and film‐forming properties (Oladzadabbasabadi, Mohammadi Nafchi, Ariffin, et al. 2022). However, the use of chitosan in food packaging is often limited by its inherent brittleness, low tensile strength, and limited flexibility, which reduce its mechanical durability under practical handling and storage conditions. Therefore, chitosan can be combined with other polymers (Al‐Maqtari et al. 2022). Research has shown that the combination of chitosan with gelatin, obtained from animal collagen, can create homogeneous, transparent, and compatible films, making chitosan film a suitable option for the preparation of intelligent films (Li et al. 2024). Another effective way to enhance the barrier and mechanical properties of chitosan films is the incorporation of materials with nanodimensional properties (Wang et al. 2023). Metal and metal oxide nanoparticles are among the most widely used nano‐materials (Lu et al. 2022).

Anthocyanins are the most interesting natural pigments in intelligent films and are sensitive to pH changes. These pigments are soluble in water, classified as secondary metabolites of plants, and fall under the category of polyphenols (Xiang et al. 2024). Anthocyanins exhibit different chemical structures at varying pH levels, and each of these structures corresponds to a distinct color, which is the reason for the color change of intelligent films containing anthocyanins at different environmental pHs (Zheng et al. 2024). Anthocyanins possess other notable features, including non‐toxicity, abundance, and biocompatibility (Oladzadabbasabadi, Mohammadi Nafchi, Ghasemlou, et al. 2022; You et al. 2022), as well as antioxidant and antimicrobial properties, which suggest health‐promoting effects (Zeng et al. 2023). These natural pigments can be extracted from different plant sources. Pomegranate ( *Punica granatum* L.) is widely cultivated for its fruit consumption in East Asia, the Mediterranean region, and the United States of America. Pomegranate flowers are considered medicinal plants that can help alleviate diseases such as chronic diarrhea and stomachaches. In China, these flowers are used to treat diabetes (Yisimayili et al. 2019). According to studies, the health benefits and antioxidant activity of pomegranate flowers are related to their polyphenol content, particularly anthocyanins, flavonoids, and tannins (Yuan et al. 2012). Pomegranate flowers are an excellent source of anthocyanins, and the two main groups of anthocyanins present in these flowers are pelargonidin‐3,5‐glucoside and pelargonidin‐3‐glucoside (Gościniak et al. 2022).

The stability of anthocyanins is significantly affected by storage and processing conditions, such as temperature, pH, oxygen, light, and enzymes, which reduce their biological efficiency and bioavailability, thereby limiting their use in the medicinal and food industries. The most efficient solution to improve the stability and bioavailability of these bioactive compounds is the encapsulation process, during which the bioactive agent is considered the core or central material and is surrounded by one or more wall materials (mainly biopolymer types) (Rashwan et al. 2023).

To the best of our knowledge, this is the first study to develop a smart, biodegradable film incorporating both zinc oxide nanoparticles and pomegranate flower anthocyanin extract nanocapsules (PFEN) into a chitosan/gelatin matrix for the purpose of monitoring fish freshness. This unique combination aims to enhance the film's functional properties while providing a visually responsive indicator of spoilage, addressing both food safety and environmental sustainability concerns. This research aims to investigate the effect of pomegranate flower extract nanocapsules on the physicochemical, barrier, mechanical, optical, and functional properties of films based on chitosan/gelatin/nano ZnO, as well as the possibility of using produced films as an indicator sensitive to pH changes to display the freshness of fish fillets during cold storage.

## 2 Materials and Methods

### 2.1 Materials

Arabic gum, maltodextrin (DE = 20), Tween 40, cold‐water fish gelatin (bloom value ~240), chitosan, glycerol, nano zinc oxide (ZnO) with a particle size range of 20–40 nm, tryptone soy agar, and all other analytical‐grade chemicals were procured from Merck (Darmstadt, Germany). The microbial strains *Escherichia coli* ATCC 43996 and *Staphylococcus aureus* ATCC 25923 were obtained from the Scientific and Industrial Research Organization of Iran (Tehran, Iran).

### 2.2 Preparation of Pomegranate Flower Extract Nanocapsules (PFEN)

#### 2.2.1 Extraction of Pomegranate Flower Anthocyanins

Pomegranate flowers were washed and rinsed, and after drying for 3 days in the dark at room temperature, they were ground and sieved (200 μ mesh). Then, 5 g of this powder was mixed with 100 mL of 50% ethanol solvent, stirred for 1 h at 500 rpm, and filtered using Whatman filter paper (No. 4). The resulting anthocyanin extract was poured into a glass container with a lid covered with aluminum foil and kept at refrigerator temperature until the next use (Hematian et al. 2023).

#### 2.2.2 Encapsulation of Pomegranate Flower Extract

To prepare pomegranate flower extract nanocapsules (PFEN), 30 g of a mixture of maltodextrin (DE = 20) and Arabic gum (50:50, w/w) was dissolved in 100 mL of distilled water and placed in a rotating hot water bath overnight until the polymer molecules were completely hydrated. Tween 40 (1% w/w) was added as a surfactant and stirred for 1 h. Pomegranate flower extract was then mixed with the wall material solution in a 1:10 ratio and homogenized for 15 min at 129 *g* at room temperature. The mixture was subsequently sonicated using a probe ultrasound (UPT150, Iran) at 20 kHz for 30 min (5‐min cycles) to reduce particle size (Parvez et al. 2022). The emulsion was dried using a spray dryer (Dorsa Tech, Iran) under the following conditions: inlet temperature 120°C, outlet temperature 88°C, feed rate 6.6 mL/min, pump speed 20% (~5 mL/min), nozzle diameter 5–8 mm, atomization pressure 1–8 bar, drying air flow 42–45 L/h, and aspirator setting 90%. The particle size distribution determined by DLS showed a polydispersity index (PDI) of 0.275, indicating uniform particles. The encapsulation efficiency (EE) was calculated based on anthocyanin content before (386.93 ± 3.27 mg/g) and after (356.87 ± 2.94 mg/g) encapsulation, resulting in an EE of ~92.2%. The particle recovery yield was 68.5%.

#### 2.2.3 Characterization of PFEN

The total anthocyanin content of the nanocapsules was measured using the differential pH method, and cyanidin‐3‐glucoside was used as the reference anthocyanin (Wang et al. 2022). Dynamic light scattering (DLS) analysis was employed to determine the mean particle size and zeta potential of PFEN. The nanocapsules were diluted with deionized water in a 1:100 ratio (Fierri et al. 2023). The PFEN morphology was studied using a scanning electron microscope (SEM; Tescan Vega3, Tescan Co., Czech Republic) at an accelerating voltage of 10 kV. To determine the encapsulation efficiency of the nanocapsules, the anthocyanin content was measured before and after the encapsulation process, and the efficiency was obtained through the following equation (Fang et al. 2020):

$$\begin{array}{c} \text{Encapsulation efficiency}\;\left(\%\right)\\ \;=\frac{\text{Total anthocyanin content of nanocapsules}}{\text{Initial total anthocyanin content}}\times 100 \end{array}$$

#### 2.2.4 Color Sensitivity of PFEN at Different pH

The PFEN solutions with varying levels of pH (3, 4, 5, 6, 7, 8, 9, 10, and 11) were prepared, their images were recorded, and the UV–Vis spectra of these solutions were analyzed in the wavelength range of 400–800 nm (Amaregouda et al. 2022).

### 2.3 Preparation of Chitosan/Gelatin/Nano ZnO/PFEN Films

To prepare the films, initially on a magnetic stirrer, a solution of 2% w/v chitosan was prepared by dissolving chitosan (9 g) in 1% v/v acetic acid (300 mL) for 6 h at 500 rpm. Then, a 4% w/v fish gelatin solution was prepared and homogenized. The chitosan and gelatin solutions were mixed in a 3:1 (v/v) ratio for 30 min. Then, 1% nano ZnO, based on dry matter, was added to 100 mL of the film solution, and 30% w/w glycerol was added. Stirring was performed for 15 min at 500 rpm. To prepare the films containing PFENs, the nanocapsules were added to the film‐making solutions at concentrations of 1%, 2%, and 3% w/v, and stirring was performed under the same conditions. After that, the film‐making solutions (15 mL) were poured onto glass petri dishes and dried at room temperature for 2 days. The control film was also prepared as above without nano ZnO and PFEN. The dried films were separated from the petri dishes, placed in plastic ziplock bags, and stored at 70% relative humidity and 30°C until the next use (Kumar et al. 2020).

### 2.4 Characterization of PFEN Films

#### 2.4.1 Investigating the Morphology of Films

The morphology of the films was examined using an SEM (TESCAN Vega3, TESCAN Co., Czech Republic) at an accelerating voltage of 10 kV. The films were glutted onto the substrate and coated with gold before imaging (Yan et al. 2021).

#### 2.4.2 Investigating the FTIR Spectra of Films

To investigate the chemical reactions between the film components, Fourier Transform Infrared Spectroscopy (FTIR; Bruker, IFS‐48, Germany) was employed in the range of 4000–400 cm^−1^ (Ebrahimi et al. 2022).

#### 2.4.3 Determining the Thickness and Mechanical Properties of Films

The thickness of the films was measured using a digital micrometer (QLR IP54, America) at five points on the films and reported in μm. The mechanical properties of the films, including tensile strength (TS) and elongation at break point (EAB), were investigated using an Instron texture analyzer (model 6025, England). The films were cut into strips with dimensions of 1 cm × 8 cm. The crosshead speed and initial grip distance were considered 50 mm/min and 40 mm, respectively. The TS and EAB of the films were obtained through the following equations (Lau et al. 2024):

$$\mathrm{TS}\;\left(\mathrm{MPa}\right)=\frac{\text{Maximum force to break the film}}{\text{Wide}\times \text{Thickness}}$$

$$\mathrm{EAB}\;\left(\%\right)=\frac{\text{Increased distance between}\;\mathrm{two}\;\text{grips}}{\text{Initial distance between}\;\mathrm{two}\;\text{grips}}\times 100$$

#### 2.4.4 Investigating Water Solubility (WS) and Water Contact Angle (WCA) of Films

First, the film samples were cut into 30 mm × 20 mm pieces, dried in an oven (Memmert, Germany) at 80°C for 24 h, and then weighed. After that, the films were immersed in deionized water (40 mL) at room temperature and reweighed after drying under the previous conditions. Through the following equation, the WS values of the films were obtained (Fernández‐Marín et al. 2022):

$$\mathrm{WS}\;\left(\%\right)=\frac{\text{Initial weight}-\text{Final weight}}{\text{Initial weight}}\times 100$$

The amount of surface wetting of the films was determined by measuring the contact angle of a water drop on the film's surface. The Amcap v3.0 software, equipped with a video camera, was used for this purpose. The films were cut into 20 mm × 20 mm squares and mounted on the holder. Distilled water was then poured onto the surface of each film through a micro‐syringe (Amaregouda et al. 2022).

#### 2.4.5 Evaluating the Water Vapor and Oxygen Permeability of Films

To investigate the water vapor permeability (WVP) of the films, the ASTM E96/E96M‐24a Standard method was employed (Rachtanapun et al. 2021). In this way, the films were initially conditioned for 2 days at a relative humidity of 52%. Then, they were sealed to the openings of the test cups (6 cm diameter) using silica gel. The cups were placed in a desiccator with a relative humidity of 52% and at room temperature. They were weighed using a digital scale (Sartorius, CP153, US) at different intervals until a constant weight was achieved. Through the slope of the line obtained from the regression equation (Δ*W*), the defined time interval (Δ*t*), and film area (A), the water vapor transfer rate (WVTR) was obtained. After that, the WVP of the films was calculated through the following equation:

$$\text{WVTR}=\frac{\Delta W}{\Delta t\times A}$$

$$\mathrm{WVP}=\frac{\text{WVTR}\times \text{Film thickness}}{\Delta P}$$

The oxygen permeability (OP) of the films was measured using the MoconOxtran 2.21, and the OP was determined using the WinPermTM permeability software in conjunction with the ASTM D3985‐22 standard method.

#### 2.4.6 Determining the Color, Opacity, and UV–Vis Light Transmission of Films

The color indices of the films, including lightness (L*), red/green (a*), and yellow‐blue (b*), were determined using a colorimeter (Minolta, China), and the following equation was used to calculate the total color differences (Δ*E*) of the films (Yan et al. 2021):

$$\Delta E=\sqrt{{\left(\Delta L\right)}^{2}+{\left(\Delta a\right)}^{2}+{\left(\Delta b\right)}^{2}}$$

A UV–Vis spectrophotometer (UV‐550; Jasco, Japan) was used to determine the UV–Vis transmittance and opacity of the films. The films were cut into 2 cm × 1 cm strips and scanned in the 300–750 nm wavelength range. To determine the opacity, the absorbance of each film was measured at 600 nm, and the opacity was calculated by dividing the absorbance by the film thickness (Huang et al. 2021).

#### 2.4.7 Examination of X‐Ray Diffraction Patterns (XRD) and Thermal Stability of Films

An X‐ray refractometer (DME, E200‐95, Germany) was used under operating conditions of 40 kV and 40 mA, with a range of 5°–60°, to investigate the crystal patterns of the films (Li et al. 2024). The thermal stability of the films was examined using a thermogravimetric analyzer (TGA; LINSEIS, PT1000, Germany) with a heating speed of 10°C/min and a temperature range of 20°C–600°C (Yi et al. 2023).

#### 2.4.8 Investigating the Antioxidant Activity of Films

The DPPH free radical scavenging method was used to investigate the antioxidant activity of the films (Guo et al. 2022). Initially, 20 mg of each film was dissolved in a 0.2 mM ethanolic solution of DPPH (4 mL). After 30 min at room temperature in the dark, the absorbance of the solutions was recorded at 517 nm. Finally, the DPPH radical scavenging of the films was calculated through the following equation and by the absorbance of the sample solution (*A*_s_) and the control solution (*A*_c_):

$$\text{DPPH}\;\left(\%\right)=\frac{A_{c}-A_{s}}{A_{c}}\times 100$$

#### 2.4.9 Investigating the Antibacterial Activity of Films

The agar diffusion disc method was used to investigate the antibacterial activity of the films against *E. coli* and *S. aureus* . Each of the bacterial suspensions was spread uniformly on the tryptic soy agar culture medium using a sterile 100 μL swab (containing approximately 10^5^ CFU/mL of bacteria). After that, films with a diameter of 2.5 cm were prepared. The films were then placed on culture medium inoculated with bacterial suspensions, and the plates were incubated at 37°C for 24 h in an incubator. After this time, the diameter of the growth inhibition zone was measured using a caliper and expressed as the antibacterial activity of the films (Zhang, Wang, et al. 2019).

#### 2.4.10 Investigating the Color Sensitivity of Films to pH Changes

To investigate the color sensitivity of the films to changes in pH, the films were cut into 20 mm × 20 mm pieces and immersed for 10 min in buffer solutions prepared with 0.1 mol/L hydrochloric acid or sodium hydroxide solutions, having a pH range of 3–11. The color indices of the films were then measured by a colorimeter (Huang et al. 2021).

### 2.5 Application of Intelligent Films to Monitoring the Freshness of Fish Fillets

Fresh rainbow trout fish were purchased from the local market and transported to the laboratory under sterile conditions and on ice. After separating the head and skin and draining the abdomen, the fish was washed and cut into approximately 15 g. The fillets were transferred to petri dishes, and the intelligent film containing 3% PFEN was attached to the inner surface of the petri dish lid with glue. The petri dishes were stored at a refrigerator temperature (4°C) for 7 days, and the TVB‐N of the fillets (determined by the micro‐Kjeldahl method) and the Δ*E* of the film (measured using a colorimeter) were recorded daily (Ekrami et al. 2022).

### 2.6 Statistical Analysis

All experiments were conducted in triplicate (*n* = 3), and data are reported as mean ± standard deviation. Statistical comparisons were performed using one‐way analysis of variance (ANOVA) followed by Duncan's multiple range test at a significance level of *p* < 0.05. Statistical analyses were conducted using SPSS software (version 22, IBM, USA).

## 3 Results and Discussion

### 3.1 Pomegranate Flower Extract and PFEN Characterization

**FIGURE 1:**
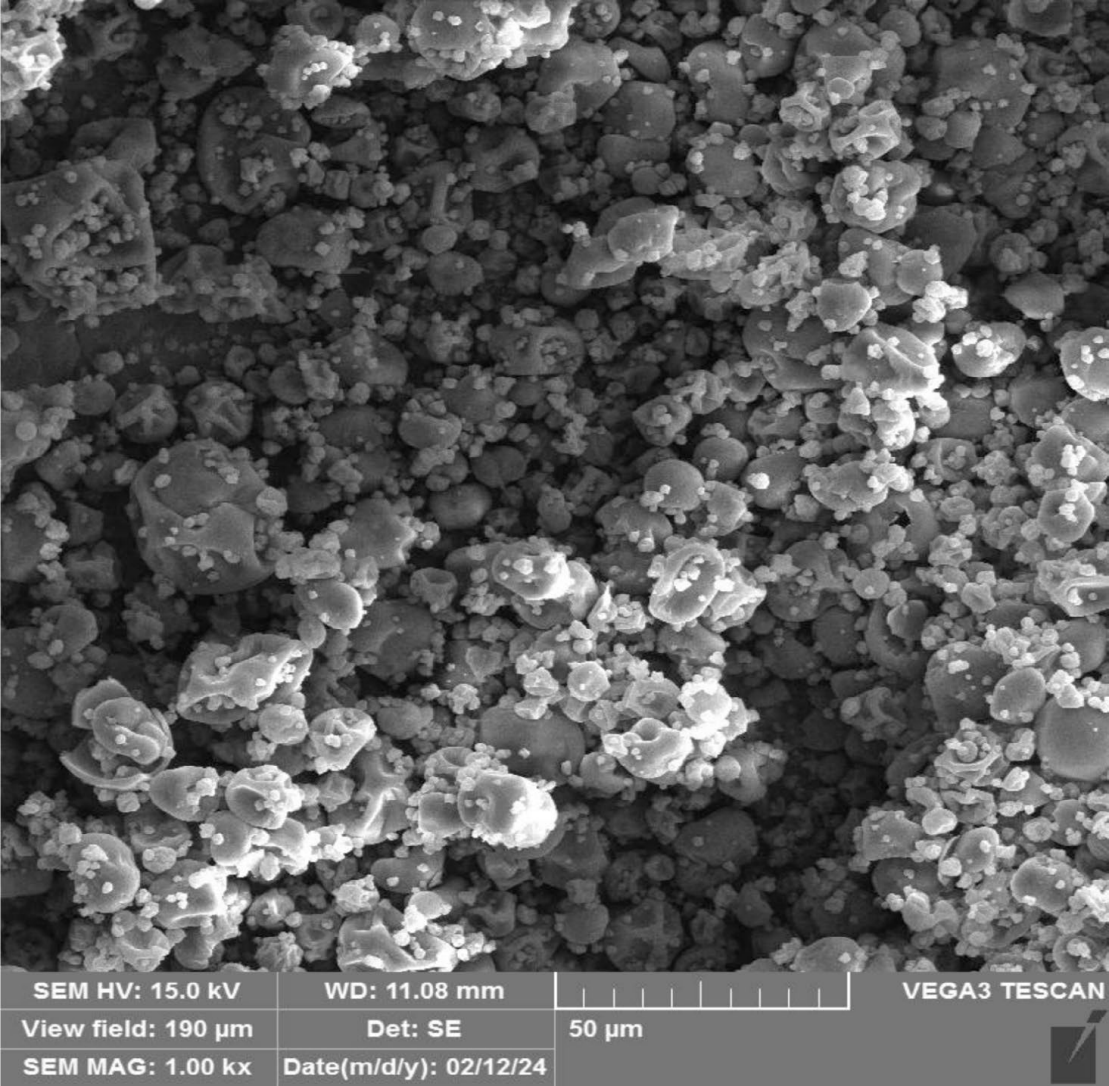
SEM image of pomegranate flower extract nanocapsules (PFEN).

The PFEN produced in this research contained 356.87 ± 2.94 mg/g of total anthocyanin. The encapsulation efficiency, mean particle size, and zeta potential of these nanocapsules were 92.55% ± 1.69%, 171.60 ± 3.15 nm, and −33.42 ± 0.73 mV, respectively. These nanocapsules exhibit high microencapsulation efficiency and a small particle size. They are in nanometer dimensions, and their surface charge is negative due to high zeta potential values; they show good stability in colloidal systems. An SEM microscope was used to investigate the morphology and microstructure of PFEN, and its image is shown in Figure 1. This image shows that the PFEN prepared by maltodextrin‐Arabic gum coating and spray drying was almost spherical with irregular and concave surfaces, and little aggregation was observed among the nanoparticles.

### 3.2 Color Response of PFEN to the pH Variation

**FIGURE 2:**
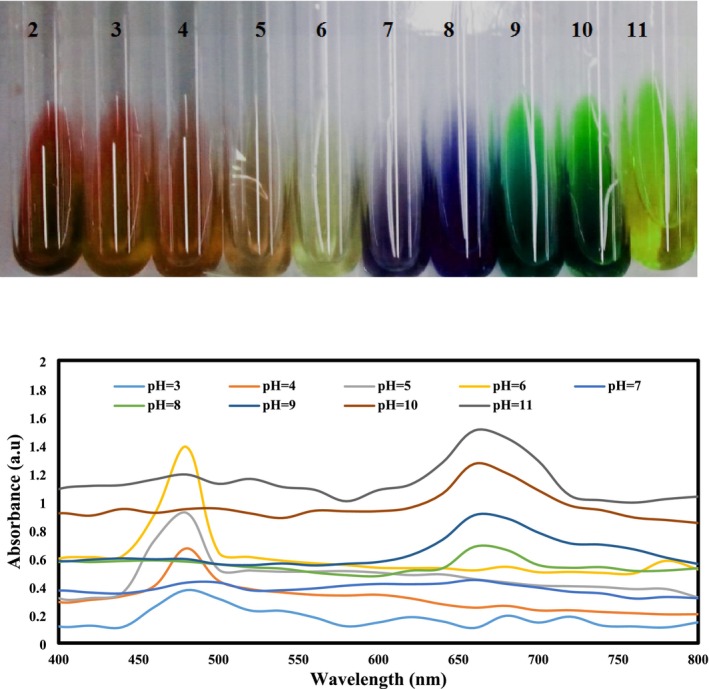
UV–Vis absorption spectra of PFEN solutions at different pH values (3–11) in the range of 400–800 nm. Increasing pH shifts the absorption peak from ~480 nm (red) to ~660 nm (green‐blue), indicating anthocyanin structural changes.

To determine the optical properties of PFEN solutions in response to pH changes, the color and UV–Vis spectra of PFEN solutions at various pH levels were investigated. The results of the color change investigation (Figure 2) showed that the color of the solutions changed clearly in response to changes in their pH. At very acidic pH around pH = 2–4, the color of solutions containing nanocapsules was in the red‐pink range, and at pH = 6, it was almost colorless and slightly yellow. At pH levels of 7 and 8, the solution's color was blue; at higher pH levels, it turned green. The color change of PFEN solutions due to changes in the pH of the buffer solution is related to the structural changes of anthocyanins at different pH levels. At a pH of 3, anthocyanins are present in a structural form as flavylium cations, which creates a red color. In the pH range of 4–6, a purple quinoidal base structure is observed, characterized by a purple‐pink color. At pH in the range of 7–9, the blue quinoidal base structure is observed, and at pH around 10, the chalcone structure is visible, which is green (Chen et al. 2021; Zhang, Liu, et al. 2019). The results of the UV–Vis spectrum investigation of anthocyanin PFEN at different pH levels are shown in Figure 2. The UV–Vis absorbance of the extract nanocapsules varied with pH; as the pH increased, a noticeable bathochromic shift (red shift) occurred, and absorbance intensity also increased, indicating structural changes in the anthocyanin compounds. While the samples with pH in the acidic range had a peak at a wavelength of 480 nm, the samples with pH in the alkaline range had a peak shifted to an approximate wavelength of 660–680 nm. This shift of the peak is related to the color change of the film from red‐pink to blue‐green, which is consistent with the results obtained by Wang et al. (2023) and Amaregouda et al. (2022).

### 3.3 Intelligent Films Characterization

#### 3.3.1 Morphology of Films

**FIGURE 3:**
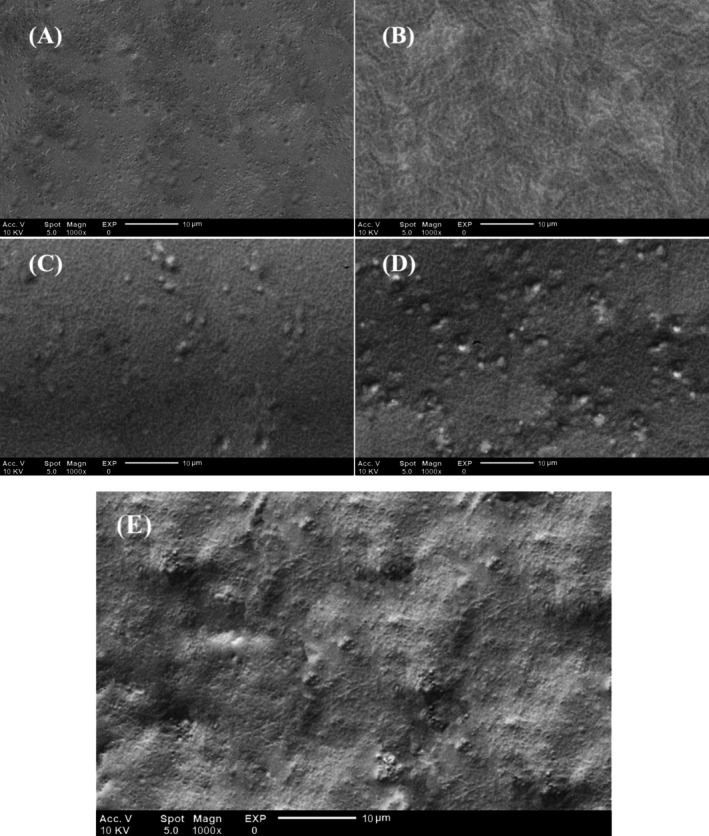
SEM images of the surface morphology of the films: (A) control; (B) ZnO + PFEN0%; (C) ZnO + PFEN1%; (D) ZnO + PFEN2%; (E) ZnO + PFEN3% at 1000× magnification. Scale bars represent 10 μm. Encapsulated particles are spherical and evenly distributed.

The surface morphology of bionanocomposite films based on chitosan and gelatin was investigated using an SEM microscope, and its images are presented in Figure 3. As the pictures show, the control chitosan/gelatin film had a dense, uniform, and smooth structure. Still, adding 1% ZnO nanoparticles, due to their filler role and filling the empty spaces of the film matrix, made the structure of the produced films denser and more uniform. The concentration of PFEN affected the morphology of the films. Films containing extract nanocapsules also had a high density. Still, at the 1% and 2% levels, a slight roughness was observed. At the 3% level, more pronounced roughness and slight cracking were noted, which is related to the accumulation of part of the extract nanocapsules at the top level in the film structure. Wang et al. (2023) observed in their research that the incorporation of eggplant anthocyanins into the chitosan film/chitin nanofiber caused surface roughness in the produced films, with the highest roughness reported at a 4% extract level. According to the research by Jiang et al. (2022), the addition of walnut peel extract to a guar gum/chitosan film at a 4% concentration resulted in a rougher film surface. The densification of the chitosan/zein film structure after adding the red radish anthocyanin extract was also observed in the research by Yi et al. (2023). Lu et al. (2022) reported the enhancement of the smooth and uniform structure of gelatin/chitosan films containing ZnO nanoparticles and anthocyanins from black peanut seed coats.

#### 3.3.2 FTIR Spectra of Films

**FIGURE 4:**
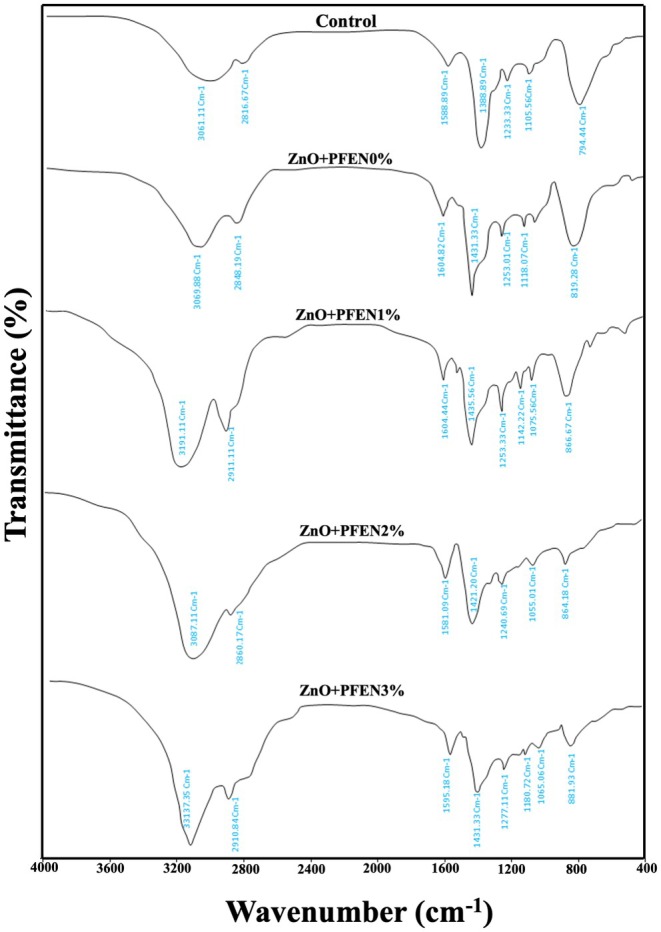
FTIR spectra of bionanocomposite films based on chitosan/gelatin containing nano ZnO and different levels of PFEN. Major peaks correspond to O—H stretching (~3300 cm^−1^), C=O stretching (amide I, ~1650 cm^−1^), N—H bending (amide II, ~1540 cm^−1^), and C—O stretching (~1040 cm^−1^).

FTIR spectroscopy was used to investigate the chemical bands of bionanocomposite films based on chitosan/gelatin containing ZnO nanoparticles and different levels of anthocyanin PFEN, and the resulting spectra are presented in Figure 4. In the control film, peaks in the area of 3061 cm^−1^ (stretching O—H groups), 2816 cm^−1^ (A amid stretching N—H), 1588 cm^−1^ (stretching C—C), 1388 cm^−1^ (stretching C—O), 1233 cm^−1^ (symmetric stretching C—O—C), and 1105 cm^−1^ (O—H glycerol) were observed (Kumar et al. 2020; Lu et al. 2022). The same peaks were also observed in the spectrum related to the chitosan/gelatin film containing ZnO nanoparticles, and partial displacement was observed in the peaks, which is related to the reactions between the film matrix and the used nanoparticles. Similar to these results, Kumar et al. (2020) also did not observe the creation of a new peak in the FTIR spectrum of gelatin/chitosan films after the incorporation of ZnO nanoparticles. In their research, just partial displacement of some peaks was reported. The results also showed that the addition of different levels of PFEN did not create a new peak in the FTIR spectrum of the films, and only a partial displacement of the peaks and broadening of the peak in the area of 3000–3300 cm^−1^ was observed, which is related to the hydroxyl groups of the extract. The peaks of 1604 and 1435 cm^−1^ in the film containing 1% PFEN, the peaks of 1581 and 1421 cm^−1^ in the film containing 2% PFEN, and the peaks of 1595 and 1431 cm^−1^ in the film containing 3% PFEN also indicate the vibrations of C=C bands of the aromatic ring, and peaks in the area of 1075 cm^−1^ in the film containing 1% PFEN, peaks in the area of 1055 cm^−1^ in the film containing 2% PFEN, and peaks in the area of 1065 cm^−1^ in the film containing 3% PFEN also show the bending vibrations of the C‐H bonds of the aromatic ring of the extract (Lu et al. 2022). In general, the addition of ZnO nanoparticles and anthocyanin PFEN to the chitosan/gelatin‐based film resulted in no new peak being observed, indicating good compatibility between the film matrix and the additives used. The partial displacement of the peaks in the FTIR spectrum of the chitosan/gelatin/nano ZnO film after the incorporation of PFEN is due to the reaction between the functional groups of the extract and the film matrix. Alizadeh Sani et al. (2022) reported the establishment of electrostatic and hydrogen bonds between barberry anthocyanin extract and TiO_2_ nanoparticles with k‐carageenan‐gelatin‐based film polymer matrix. In the research by Chen et al. (2021), only hydrogen bonds were established between the red cabbage anthocyanin extract and the chitosan/cellulose nanocrystal film matrix, indicating a physical mixing. The research of Li et al. (2024) also confirmed the establishment of hydrogen bonds between anthocyanins of butterfly pea flower extract and chitosan/gelatin film matrix.

#### 3.3.3 Thickness and Mechanical Properties of Films

The thickness values of bionanocomposite films based on chitosan/gelatin are shown in Table 1. The control film had the lowest thickness (91.22 μm), and incorporating ZnO nanoparticles into the films significantly increased the thickness to 93.72 μm. Adding PFEN and increasing its level also increased the film thickness (*p* < 0.05). The thickness of the films containing PFEN ranged from 96.48 to 99.10 μm. The increase in film thickness resulting from the incorporation of PFEN is directly related to the increase in solid content of the film‐forming solutions. Other researchers also observed these results. For example, Hematian et al. (2023) reported an increase in the thickness of gelatin films due to the addition of *Solenostemon* anthocyanin extract. Lu et al. (2022) also demonstrated an increase in the thickness of gelatin/chitosan films after incorporating ZnO nanoparticles and anthocyanins from black peanut seed coating. Gasti et al. (2021) also revealed that the thickness of films based on chitosan/methylcellulose containing *Phyllanthus reticulatus* anthocyanin extract was significantly higher than that of the control film and attributed this increase in thickness to the increased solids content resulting from the presence of anthocyanins.

**TABLE 1:** Thickness, solubility, WVP, and OP of chitosan/gelatin films containing nano ZnO and different levels of PFEN.

Films	Thickness (μm)	Solubility (%)	WVP (10^−11^ g m/m^2^ day)	OP (cc/m day atm)
Control	91.22 ± 1.04^d^	25.48 ± 0.31^a^	6.37 ± 0.24^a^	2.74 ± 0.03^a^
ZnO + PFEN0%	93.72 ± 0.85^c^	21.96 ± 0.54^b^	4.10 ± 0.13^b^	1.97 ± 0.08^b^
ZnO + PFEN1%	96.48 ± 0.91^b^	21.37 ± 0.57^bc^	3.62 ± 0.17^c^	1.66 ± 0.04^c^
ZnO + PFEN2%	97.61 ± 1.12^ab^	20.59 ± 0.46^cd^	3.17 ± 0.05^d^	1.39 ± 0.11^d^
ZnO + PFEN3%	99.10 ± 0.88^a^	20.13 ± 0.35^d^	2.94 ± 0.18^d^	1.20 ± 0.07^e^

*Note:* Values are mean ± SD (*n* = 3). Different superscript letters indicate significant differences (*p* < 0.05, one‐way ANOVA, *F*(4, 10) = 12.37).

Abbreviations: OP, oxygen permeability; PFEN, pomegranate flower extract nanocapsules; WVP, water vapor permeability.

**FIGURE 5:**
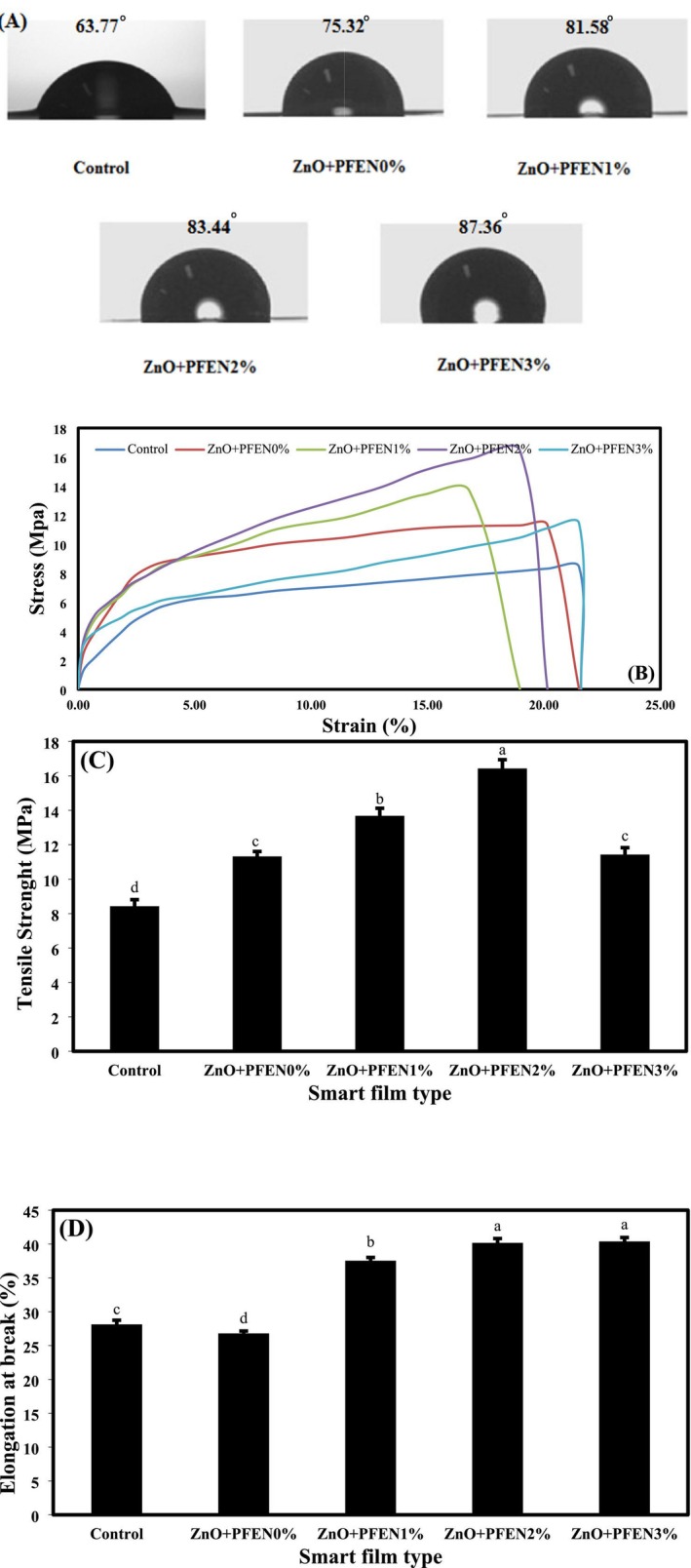
(A) Water contact angle (°), (B) stress–strain curves, (C) TS (MPa), and (D) EAB (%) of bionanocomposite films based on chitosan/gelatin containing nano ZnO and different levels of PFEN. Bars are mean ± SD (*n* = 3). Different superscript letters indicate significant differences (*p* < 0.05, one‐way ANOVA, *F*(4, 10) = 12.37).

Stress–strain curves and values of the tensile strength (TS) and elongation at break point (EAB) of bionanocomposite films based on chitosan/gelatin are shown in Figure 5. The values of TS and EAB of the control film were 8.43 MPa and 28.13%, respectively. With the incorporation of ZnO nanoparticles, the tensile strength (TS) of the films increased significantly, reaching 11.32 MPa. In contrast, the EAB value of the films decreased, reaching 26.80%, and the flexibility of the films also decreased. In the research conducted by Rahman et al. (2018), which is consistent with the results of the present study, adding 1% ZnO nanoparticles to chitosan‐based films increased the tensile strength (TS) of the films compared to the control group. These researchers found that the improvement in the mechanical strength of the films due to the incorporation of nanofillers is attributed to the nanoparticles being placed between the polymer chains of the film matrix, which exhibits a cross‐linking effect. However, some researchers have reported a decrease in the mechanical strength of the films after incorporating ZnO nanoparticles, which they attribute to the formation of weak hydrogen bonds between the polymer matrix and the nanoparticles. Adding PFEN and increasing its level from 0% to 2% significantly increased the TS and EAB of the films (*p* < 0.05). However, with the increase in the nanocapsule level from 2% to 3%, the TS of the films decreased, but no significant change was observed in the EAB of the films.

The average values of TS and EAB of films containing PFEN were 11.43–16.43 MPa and 37.54%–40.39%, respectively. In general, using lower levels of PFEN improved the mechanical strength of the films due to their denser structure; however, at high levels of PFEN, the tensile strength (TS) of the films decreased due to the partial accumulation of nanoparticles. The researchers stated that establishing hydrogen bonding between the extract and the biopolymer film can densify the film's structure and enhance its mechanical strength (Jiang et al. 2020). On the other hand, anthocyanins can act as a plasticizer, thereby enhancing the flexibility of films (Alizadeh Sani et al. 2022). In their research, Dong et al. (2023) observed an improvement in the mechanical strength of biopolymer films due to the incorporation of purple sweet potato anthocyanin extract and quercetin nanocapsules. Zeng et al. (2023) reported that the addition of black rice extract to chitosan/pectin film improved the mechanical properties of the produced films. Another study observed an increase in the mechanical strength and elongation percentage of gelatin/chitosan films after incorporating ZnO nanoparticles and anthocyanins from black peanut seed coating (Lu et al. 2022).

#### 3.3.4 Water Solubility (WS) and Water Contact Angle (WCA) of Films

Water solubility (WS) indicates the film's stability in the presence of water. The water solubility values of bionanocomposite films based on chitosan/gelatin are shown in Table 1. The control film had the highest WS (25.48%). Chitosan has hydrophilic groups in its structure, including hydroxyl groups and amino groups, and, in general, films based on this biopolymer have high water solubility (Zhang, Liu, et al. 2019). Gelatin is also soluble in water. By incorporating ZnO nanoparticles, the solubility of the films decreased significantly, reaching 21.96%. The addition of ZnO nanoparticles decreased the work of adhesion (WS) of the produced films through intermolecular reactions with the film matrix and increased the density of the film structure.

On the other hand, ZnO nanoparticles are insoluble in water, which increases the film's hydrophobicity. These observations were also reported in the research conducted by Wang et al. (2023). Adding PFEN and increasing its level also decreased the WS of the films (*p* < 0.05). The average values of WS of films containing PFEN were in the range of 20.13%–21.37%. The decrease in the water solubility (WS) of the films due to the incorporation of anthocyanin extracts is likely related to the formation of hydrogen bonds between the hydroxyl groups of anthocyanins and phenolic compounds within the film matrix, which reduces the free hydroxyl groups' ability to bind with water. In this way, the WS of the films decreases. Lu et al. (2022) observed a decrease in the WS of gelatin/chitosan films after incorporating ZnO nanoparticles and black peanut seed coating anthocyanins. The reduction in the WS of chitosan films after combining the extracts of eggplant and black plum peel was also observed in studies conducted by Wang et al. (2023), Zhang, Liu, et al. (2019), and Zhang, Wang, et al. (2019).

The WCA of films is a test used to investigate the surface wetness of films and their surface hydrophobicity (Gao et al. 2022). The WCA values of bionanocomposite films based on chitosan and gelatin are shown in Figure 5. The control film had the lowest water contact angle (WCA) of 63.77°. Films with a WCA greater than 65° are considered hydrophobic surfaces, while values < 65° indicate the surface hydrophilicity of the films (Gasti et al. 2021). The surfaces of chitosan/gelatin films are only hydrophilic. The hydrophilicity of the chitosan film surfaces has also been demonstrated in research conducted by Ezati and Rhim (2020). By incorporating ZnO nanoparticles, the WCA of the films increased significantly, reaching 75.32°, and the hydrophobicity of the films also improved. Adding PFEN and increasing its level also significantly increased the WCA of the films (*p* < 0.05). The average WCA values of films containing nanocapsules were in the range of 81.58°–87.36°. These observations are likely due to the phenolic compounds and anthocyanin extract establishing hydrogen bonds with the hydrophilic groups of chitosan, thereby reducing the free hydroxyl groups available on the surface of the film to bond with water and increasing the hydrophobicity of the chitosan film's surface (Wang et al. 2023). Creating roughness on the surfaces of films can also increase their hydrophobicity (Hou and Yan 2021). The research of Wang et al. (2023) reported an increase in WCA of chitosan/chitin nanofiber films after the incorporation of eggplant anthocyanin extract. An increase in the WCA and a decrease in the hydrophilicity of chitosan/zein films were also observed in another study (Yi et al. 2023) due to the incorporation of red radish anthocyanin extract.

#### 3.3.5 Water Vapor (WVP) and Oxygen Permeability (OP) of Films

The WVP values of bionanocomposite films based on chitosan and gelatin are shown in Table 1. The control film had the highest WVP (6.37 × 10^11^ g m/m^2^ day), and with the incorporation of ZnO nanoparticles, the WVP of the films decreased significantly, reaching 4.10 × 10^11^ g m/m^2^ day. Adding PFEN and increasing its level also reduced the permeability of the films (*p* < 0.05). The WVP values of films containing PFEN were 2.94–3.62 × 10^11^ g m/m^2^ day. The decrease in WVP of the films due to the incorporation of anthocyanin extracts can be attributed to the establishment of hydrogen bonds between the anthocyanins of the extract and the polymer matrix of the film, as a result of which, fewer free hydroxyl groups are available to water molecules. In this way, the solubility of the films in water decreases (Wang et al. 2023).

On the other hand, anthocyanins have bulky aromatic rings in their skeletal structure, making the film network denser and more difficult for water vapor to pass through (Chen et al. 2021). Nanofillers also densify the film structure, reducing the passage and movement of water vapor through the film matrix and thereby decreasing the WVP (Yu et al. 2017). Eze et al. (2022) reported an improvement in the water vapor permeability (WVP) properties of chitosan films after incorporating broken raspberry extract. Alizadeh Sani et al. (2022) showed that the combination of barberry anthocyanin extract and TiO_2_ nanoparticles could improve the stability of k‐carrageenan‐gelatin‐based films against moisture and improve the hydrophobicity of the films. In another study, red cabbage anthocyanin extracts were found to enhance the water vapor permeability (WVP) of chitosan/pullulan/chitin nanofiber films (Wu et al. 2022).

The OP packaging of films greatly impacts the shelf life of food products. The values of the oxygen permeability (OP) of bionanocomposite films based on chitosan/gelatin are shown in Table 1. The control film had the highest OP (2.74 × 10^−6^ cc/m day atm). Previous research has also demonstrated the weak gas barrier properties of chitosan (Jiang et al. 2022). By incorporating ZnO nanoparticles, the permeability of the films decreased significantly, reaching 1.97 × 10^−6^ cc/m day atm. In previous research, they also reported an improvement in the barrier properties of biopolymer films against oxygen due to the incorporation of ZnO nanoparticles, attributing this observation to the difficulty in gas movement (Babapour et al. 2021; Yadav et al. 2021). Adding PFEN and increasing its level also significantly reduced the oxygen permeability (OP) of the films (*p* < 0.05). The values of OP of films containing PFEN were in the range of 1.20–1.66 × 10^−6^ cc/m day atm. Nanoparticles can be placed in the holes of the film structure, and by reducing the number of holes, they make it more difficult for gases to pass through the film structure, thereby improving the OP and WVP of the films (Ma et al. 2022). You et al. (2022) reported that after the incorporation of anthocyanin extract from red grape skin, a decrease in the OP of k‐carrageenan films was observed. These researchers attributed their results to the reduction of empty spaces in the film, as well as the polarity of the bioactive compounds in the extracts. Since oxygen is a non‐polar compound, increasing the polarity of the films can decrease the amount of oxygen passing through them (Ma et al. 2017). Chen et al. (2021) reported that by incorporating red cabbage anthocyanin extract into the chitosan/chitin nanocrystal film structure, the WVP and OP of the films decreased significantly. Wang et al. (2023) also showed that after the incorporation of eggplant anthocyanin extract, the OP of chitosan/chitin nanofiber films decreased significantly.

#### 3.3.6 Color and Opacity of Films

The appearance of food packaging has a significant effect on consumers' acceptance of the product. The color indices of the films, including brightness (L*), green‐red (a*), blue‐yellow (b*), and total color difference (Δ*E*), were studied using the calorimeter. The values of color indices and the opacity of bionanocomposite films based on chitosan and gelatin are shown in Table 2. The control film and the film containing ZnO nanoparticles had the highest color brightness (89.84 and 90.13, respectively). With the addition of PFEN and an increase in its level, a significant decrease in the color brightness of the films was observed (*p* < 0.05). In terms of the a* index, the intensity of redness of the control sample (2.13) was lower than that of the sample containing ZnO nanoparticles (0.57), and the addition of PFEN and increasing its level caused a significant increase in the a* index of the produced films. The slight decrease in the a* value observed in the ZnO + PFEN0% film compared to the control may be attributed to the intrinsic whiteness and UV‐scattering properties of ZnO nanoparticles, which can reduce red color intensity by disrupting anthocyanin light absorption or reflectance within the film matrix. In terms of the b* index, the control sample had the lowest intensity of yellowness (8.69). The incorporation of ZnO nanoparticles, as well as PFEN, significantly increased the yellowness of the films (*p* < 0.05). With the increase in the level of PFEN, the overall color change of the films showed a significant increase. The values of L*, a*, b*, and Δ*E* for films containing different levels of PFEN were obtained as follows: 65.34–77.56, 7.80–12.24, 13.32–14.52, and 14.29–27.14, respectively. The control sample and the film containing ZnO nanoparticles had the lowest amount of opacity (1.12 and 1.17 mm^−1^, respectively), and there was no statistically significant difference between these two films. By incorporating PFEN and increasing its level from 1% to 3%, the opacity of the films increased significantly (*p* < 0.05). It ranged from 2.64 to 5.56 mm^−1^, which is attributed to the presence of anthocyanin pigments in the pomegranate extract, resulting in more colorful films.

**TABLE 2:** Color indexes and opacity of chitosan/gelatin films containing nano ZnO and different levels of PFEN.

Films	L*	a*	b*	Δ*E*	Opacity (mm^−1^)
Control	89.84 ± 1.35^a^	2.13 ± 0.16^d^	8.69 ± 0.48^d^	—	1.12 ± 0.04^d^
ZnO + PFEN0%	90.13 ± 1.58^a^	0.57 ± 0.07^e^	10.03 ± 0.26^c^	2.08 ± 0.19^d^	1.17 ± 0.08^d^
ZnO + PFEN1%	77.56 ± 0.93^b^	10.80 ± 0.19^c^	13.32 ± 0.23^b^	14.29 ± 0.61^c^	2.64 ± 0.10^c^
ZnO + PFEN2%	70.98 ± 1.12^c^	12.71 ± 0.12^b^	13.86 ± 0.37^ab^	20.97 ± 0.48^b^	3.93 ± 0.07^b^
ZnO + PFEN3%	65.34 ± 1.36^d^	15.24 ± 0.18^a^	14.52 ± 0.51^a^	27.14 ± 0.69^a^	5.56 ± 0.13^a^

*Note:* Values are mean ± SD (*n* = 3). Different superscript letters indicate significant differences (*p* < 0.05, one‐way ANOVA, *F*(4, 10) = 12.37).

Abbreviation: PFEN, pomegranate flower extract nanocapsules.

The a* value of the ZnO + PFEN0% film was lower than that of other samples. This can be explained by the *light‐scattering effect of ZnO nanoparticles*, which reduces the perceived redness by increasing opacity and diminishing the intensity of transmitted/absorbed light. Similar reductions in color parameters have been reported in nanoparticle‐loaded biopolymer films due to scattering phenomena.

**FIGURE 6:**
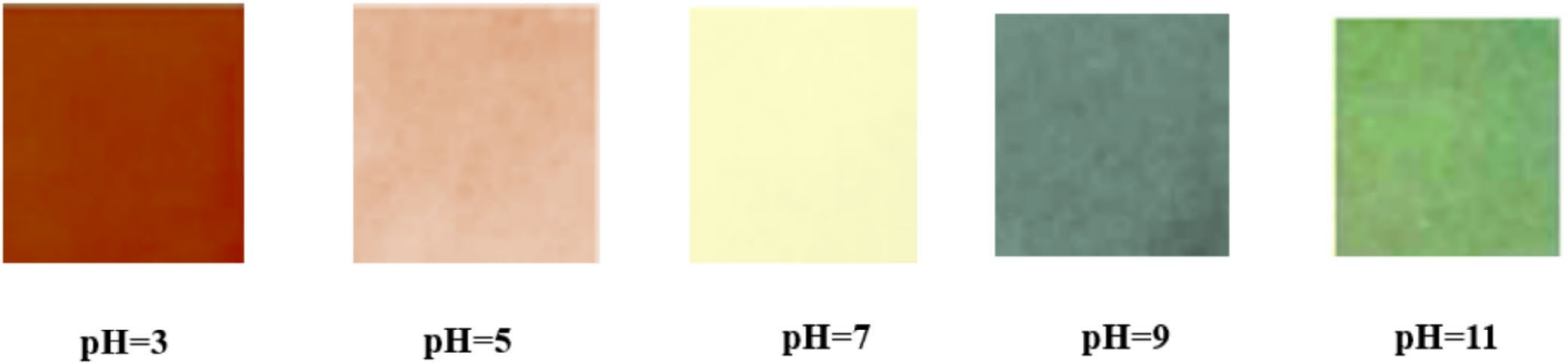
Color of bionanocomposite film based on chitosan/gelatin/nano ZnO containing 3% PFEN in different pHs.

Figure 6 illustrates the visible color of the films at various pH levels. Hematian et al. (2023) showed, in agreement with the results of the present study, that adding the anthocyanin extract of Solenostemon leaves to gelatin films decreased the color brightness of the films. The a* and b* indices increased, and the films became darker in color. Chen et al. (2021) reported that increasing the level of red cabbage anthocyanin extract in the chitosan/chitin nanocrystal film resulted in decreased color brightness and increased redness, producing darker films. You et al. (2022) also reported a decrease in brightness and an increase in the redness and opacity of biopolymer films after incorporating anthocyanin extract into red grape skin.

#### 3.3.7 UV–Vis Light Transmission From Films

**FIGURE 7:**
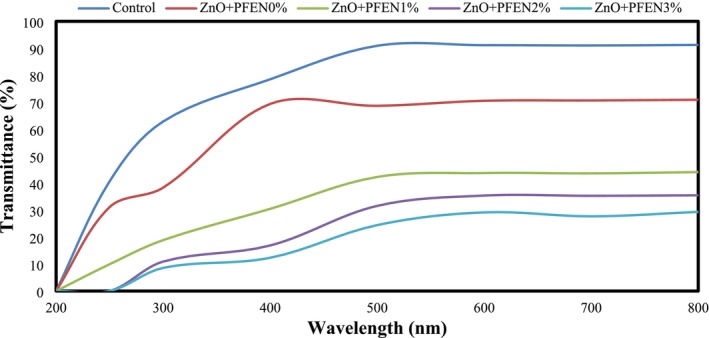
UV–Vis light transmittance from bionanocomposite films based on chitosan/gelatin containing nano ZnO and different levels of PFEN.

The evaluation of the UV–Vis light transmission percentage of food packaging films is crucial because UV light significantly contributes to the chemical degradation of food products, and films with a lower light transmission percentage exhibit a higher protective role (Gasti et al. 2021). The percentage of UV–Vis light transmission of bionanocomposite films based on chitosan/gelatin containing ZnO nanoparticles and different levels of PFEN are compared in Figure 7. As can be seen, the highest transmission of UV–Vis light was related to the control film. After adding ZnO nanoparticles to the films, a significant decrease in the percentage of UV–Vis light transmission from the films was observed, which is related to the strong absorption or scattering of UV light by ZnO nanoparticles. These observations were also consistent with the results obtained by Huang et al. (2021) and Zhang et al. (2023). The incorporation of PFEN led to a further decrease in the percentage of UV–Vis light transmission of bionanocomposite films, and increasing the level of PFEN also significantly reduced the percentage of light transmission. The developed films displayed substantial UV‐blocking efficiency, reducing UV transmittance by over 50% in the UVA and UVB regions. Similar enhancements have been observed in recent studies: films incorporating ZnO nanoparticles and anthocyanin extracts achieved UV‐A/B blocking efficiencies ranging from 40% to 70% (Boopasiri et al. 2023; Ezati et al. 2023; Sathianathan et al. 2025). These findings support the potential of ZnO–anthocyanin composites for effective UV protection in food packaging applications. The increase in the opacity of films due to the incorporation of PFEN is a reason for improving the light obstruction properties of films containing different levels of this extract.

On the other hand, research has shown that anthocyanins can absorb UV light, and their presence in biopolymer films can reduce the percentage of light transmission from produced films (Jiang et al. 2022; Kanatt 2020). You et al. (2022) also demonstrated that anthocyanins act as UV light absorbers, and their incorporation into biopolymer films can significantly enhance the light barrier properties of the films. These researchers also reported that the barrier activity of anthocyanins in the UV spectrum range was higher than that of silver nanoparticles. In contrast, silver nanoparticles showed better inhibitory properties in the visible light range. Lu et al. (2022) also observed the light‐inhibitory properties of gelatin/chitosan films after the incorporation of ZnO nanoparticles and, in particular, anthocyanins from the black peanut seed coat. Chen et al. (2021) stated that chitosan film has a weak light barrier property; however, by incorporating red cabbage anthocyanin extract into this film, the percentage of light transmission through the films decreased significantly, and the barrier properties of the film improved.

#### 3.3.8 X‐Ray Diffraction Pattern (XRD) of Films

**FIGURE 8:**
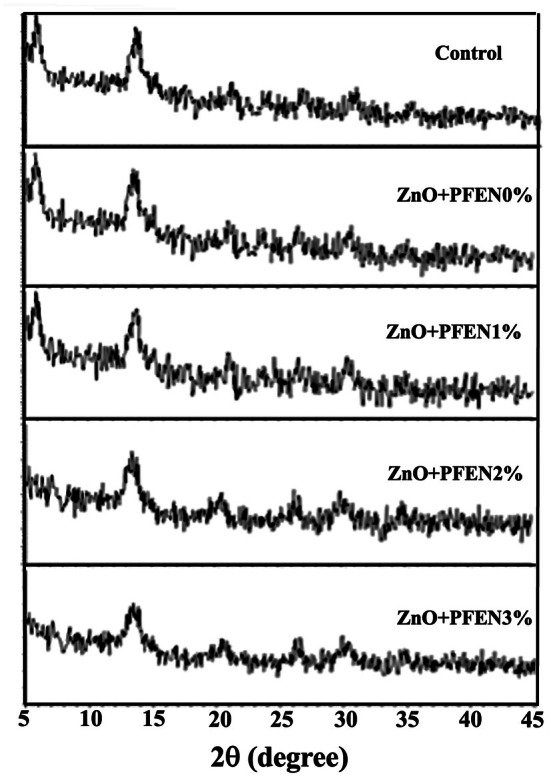
XRD patterns of bionanocomposite films based on chitosan/gelatin containing nano ZnO and different levels of PFEN.

XRD was used to investigate the structure and crystallinity of bionanocomposite films based on chitosan/gelatin containing ZnO nanoparticles and different levels of PFEN, and the resulting patterns are presented in Figure 8. The results showed that in the control film, large peaks were observed at 2θ angles of 5.84° and 14°, corresponding to crystalline hydrated structures. Peaks were also observed in the areas of 20°–30°, which are weak and related to the amorphous regions of the film structure. The spectrum of the chitosan/gelatin film as a whole indicated the crystalline hydrated structure of this film (Wang et al. 2023). As a result of incorporating ZnO nanoparticles alone or in combination with the level of 1% PFEN, no change was observed in the XRD patterns of the films; however, the addition of 2% and 3% PFEN caused a significant decrease in the peak area of 2ϴ = 5.84°, which indicates the reduction in the crystallinity of the film structure after incorporating high levels of PFEN. In agreement with these results, Chen et al. (2021) reported that by increasing the level of red cabbage anthocyanin extract in the chitosan/chitin nanocrystal film, the crystallinity of the film decreased and degradation in the film structure was observed, which is due to the formation of amorphous complexes due to the creation of hydrogen bonds between the film and the extract. These results are also consistent with those obtained by Zhang, Liu, et al. (2019), who reported a decrease in the crystallinity of the chitosan film due to the addition of purple sweet potato extract. In the research of Yi et al. (2023), the incorporation of red radish anthocyanin extract decreased the crystallinity peak of the chitosan/zein film.

#### 3.3.9 Thermal Stability of Films

**FIGURE 9:**
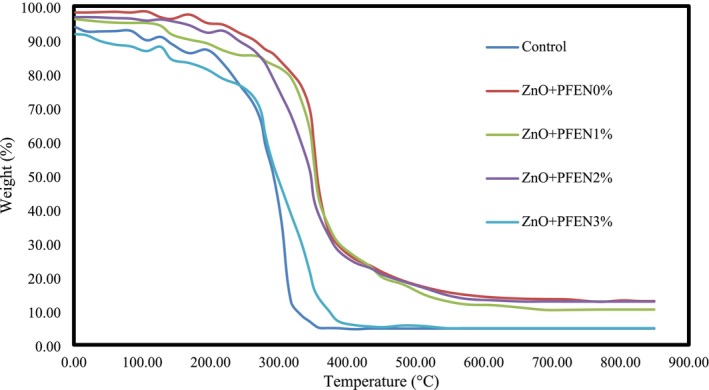
TGA thermograms of bionanocomposite films based on chitosan/gelatin containing nano ZnO and different levels of PFEN.

Thermogravimetric analysis (TGA) and differential thermogravimetric (DTG) analysis were used to evaluate the thermal stability of the films. The TGA curves (Figure 9) showed three major weight loss stages: initial moisture evaporation (~100°C) (Yi et al. 2023; Zhang et al. 2020), degradation of glycerol and low molecular weight compounds (150°C–250°C), and polymer matrix decomposition (250°C–500°C). DTG curves revealed that films containing ZnO and PFEN exhibited higher peak degradation temperatures and lower overall mass loss compared to control films, indicating improved thermal stability. The increased residual mass observed in ZnO‐ and PFEN‐loaded films may be attributed to the presence of thermally stable inorganic components (ZnO) and enhanced cross‐linking within the polymer matrix (Ren et al. 2023). Incorporation of ZnO nanoparticles into the chitosan/gelatin film can improve the thermal stability of the films and alter the degradation of the film structure at higher temperatures. The films containing PFEN also showed higher thermal stability than the control group; however, with the increase in nanocapsules, the thermal stability of the films decreased. The improving effect of ZnO nanoparticles on the thermal stability of chitosan/cellulose acetate terephthalate film was also studied by Indumathi et al. (2019). Kumar et al. (2020) reported an improvement in the thermal stability of chitosan/gelatin films after incorporating ZnO nanoparticles, attributing this effect to an increased reaction between the polymer chains. In the research by Dong et al. (2023), the thermal stability of agar‐sodium alginate films was observed when combined with quercetin nanoparticles and purple sweet potato anthocyanin extract.

#### 3.3.10 Antioxidant Activity of Films

**FIGURE 10:**
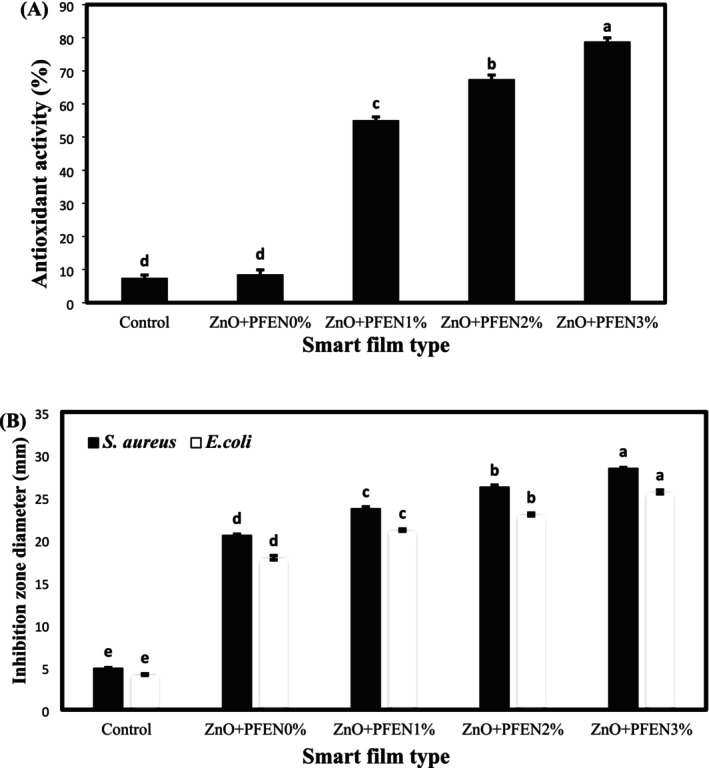
(A) Antioxidant and (B) antibacterial activity of bionanocomposite films based on chitosan/gelatin containing nano ZnO and different levels of PFEN. Bars are mean ± SD (*n* = 3). Different superscript letters indicate significant differences (*p* < 0.05, one‐way ANOVA, *F*(4, 10) = 12.37).

DPPH radical scavenging activity was employed to investigate the antioxidant properties of the films. The antioxidant activity values of bionanocomposite films based on chitosan/gelatin are shown in Figure 10a. The control sample and the film containing ZnO nanoparticles exhibited the lowest antioxidant activity (7.49% and 8.56%, respectively), with no statistically significant difference between the two films. By incorporating PFEN and increasing its level from 1% to 3%, the antioxidant activity of the films increased significantly, reaching 55.12% to 78.86%. The antioxidant activity of the films was generally dependent on the concentration of the anthocyanin extract used. The presence of phenolic and anthocyanin compounds in pomegranate flower extract is the reason for the increase in the antioxidant activity of the films after incorporating the nanocapsules of this extract, and due to the increase in the nanocapsule surface, the antioxidant activity of the films increased significantly due to the increase in the concentration of phenolic and anthocyanin compounds. Polyphenols and anthocyanins can neutralize free radicals and block oxidation chain reactions (Hao et al. 2023). The strong antioxidant activity of pomegranate flower extracts has also been observed in the research of Küçükbay and Teki̇n (2020), who found that the antioxidant activity of pomegranate flower extracts was higher than that of α‐tocopherol and BHT antioxidants. A significant increase in the antioxidant activity of films based on chitosan/chitin nanocrystals after incorporating red cabbage anthocyanin extract was also found in the research of Chen et al. (2021). Lu et al. (2022) also found that incorporating ZnO nanoparticles and anthocyanins from black peanut seed coating into gelatin/chitosan films enhanced the antioxidant activity of the produced films. Still, the antioxidant effect of anthocyanin was significantly higher than that of nano ZnO.

#### 3.3.11 Antibacterial Activity of Films

The agar disk diffusion method was used to investigate the antibacterial activity of bionanocomposite films against two pathogen strains, including *S. aureus* (a Gram‐positive bacterium) and *E. coli* (a Gram‐negative bacterium). The diameter values of the inhibition zone of bionanocomposite films based on chitosan/gelatin against two bacterial strains are shown in Figure 10b. The control sample exhibited the lowest antimicrobial activity, with inhibition zone diameters of 4.87 mm against *S. aureus* and 4.13 mm against *E. coli* , respectively. Gelatin generally lacks antimicrobial activity, and chitosan in the film‐making solution shows weak antimicrobial activity, which is related to the cationic nature of chitosan that can react with the cell membrane of pathogenic bacteria and cause an increase in the permeability of the cell membrane (Wang et al. 2023; Zhang et al. 2020). After adding ZnO nanoparticles to the films, the antibacterial activity increased significantly (*p* < 0.05). The diameter of the inhibition zone of the film containing ZnO nanoparticles without extract against *S. aureus* and *E. coli* was 20.49 and 17.87 mm, respectively. By incorporating PFEN and increasing its level from 1% to 3%, the antibacterial activity of the films against both bacterial strains was significantly improved. The diameter of the inhibition zone of the films with different levels of PFEN against *S. aureus* was in the range of 28.37–23.63 mm, and against *E. coli* was in the range of 21.11–25.60 mm. Anthocyanins can directly affect the metabolism of pathogens and increase the permeability of the bacterial cell membrane. These compounds also inhibit the action of extracellular enzymes of microorganisms, preventing the arrival of substrates necessary for bacterial cell growth and thereby exert their antimicrobial activity (Cisowska et al. 2011; Gasti et al. 2021).

The polyphenols in the extract also exert their antimicrobial activity by changing the cell wall, increasing membrane permeability, and inhibiting RNA and DNA synthesis (Wang et al. 2023). The films examined in this research generally exhibited higher antimicrobial activity against Gram‐positive bacteria compared to Gram‐negative bacteria. This observation is related to the more complex structure of Gram‐negative bacteria, as these bacteria have a thick layer of peptidoglycan surrounding their cell wall, which provides a protective effect to the bacterial cell. On the other hand, there is a difference between the metabolism and cell physiology of these two groups of bacteria, which affects the antimicrobial performance of bioactive compounds (Zhang et al. 2020). Alizadeh Sani et al. (2022) showed that the incorporation of barberry anthocyanin extract and TiO_2_ nanoparticles could improve the antimicrobial activity of k‐carageenan‐gelatin‐based films. Dong et al. (2023) also reported an increase in the antibacterial activity of agar‐sodium alginate films after incorporating sweet potato anthocyanin extract and quercetin nanoparticles prepared with chitosan. They observed the activity of these films against *S. aureus* above *E. coli* . Lu et al. (2022) observed that incorporating ZnO nanoparticles and black peanut coating anthocyanins into gelatin/chitosan films resulted in significant antimicrobial activity in the produced films. The researchers reported that the antimicrobial activity of the nanoparticles exceeded that of anthocyanins.

It should be noted that the agar diffusion assay used in this study provides only preliminary insights into antimicrobial activity and is inherently semi‐quantitative. The observed inhibition zones therefore indicate antibacterial potential but do not confirm detailed mechanisms. Previous studies have reported that ZnO nanoparticles exert antibacterial effects primarily via reactive oxygen species (ROS) generation and disruption of bacterial membranes, while anthocyanins may interact with lipid bilayers, altering permeability and protein function (Akbariazam et al. 2016; Javidi et al. 2022). Future work should incorporate more quantitative assays, including minimum inhibitory concentration (MIC), minimum bactericidal concentration (MBC), and time‐kill studies, to confirm and expand upon the preliminary results presented here.

#### 3.3.12 Color Sensitivity of Films to pH Changes

Chitosan/gelatin‐based bionanocomposite films containing ZnO nanoparticles and different levels of PFEN were placed in buffer solutions with acidic (pH = 3), neutral (pH = 7), and alkaline (pH = 11) pH levels, and changes in the color indices of the films were measured using a calorimeter. The results are presented in Table 3. As the results show, the control film and the film containing ZnO nanoparticles without extract had the highest brightness at all pH levels. As expected, no change in the color intensity of these two films was observed with the change in the pH of the buffer solution. However, after placing the films containing different levels of PFEN in solutions with varying pH levels, a significant reduction in the color brightness of these films occurred. A clear color change was also observed in the films, resulting in a nearly red‐purple color at acidic pH levels. At a neutral pH, they were yellowish, and at an alkaline pH, they were green‐blue. Films with ∆*E* above 5 exhibit clear color changes, which are easily observed (Yi et al. 2023). Since films containing PFEN at different pH levels had an ∆*E* above 5, their color changes are visible to the human eye. The results of this test generally indicate the color sensitivity of the bionanocomposite films based on chitosan/gelatin/ZnO nanoparticles containing different levels of PFEN to changes in pH, making these films suitable for use as intelligent pH‐sensitive films. Yan et al. (2021) also demonstrated that chitosan films containing butterfly pudding extract were sensitive to changes in the pH of the buffer solution. With changes in pH from 1 to 14, the color of the films shifted from purple‐pink to yellow. You et al. (2022) also found that the color of k‐carrageenan/silver nanoparticles/anthocyanin extract of red grapes films changed against changes in the pH of the buffer solution, and the color of these films was pink in the acidic pH, blue in the neutral pH, and green in the alkaline pH, but the color of the film without the extract did not change in different pHs. Yi et al. (2023) also showed that the color of chitosan/zein/anthocyanin film of red radishes depends on the pH of the buffer solution, and the color of this film in acidic pH between 3 and 2 was red‐brown, in acidic pH between 4 and 8 was brown, and in alkaline pHs 12 and 11 was yellow‐green.

**TABLE 3:** The color changes of smart chitosan/gelatin films containing nano ZnO and different levels of PFEN at different pHs.

pH	Sample	Color attributes			Δ*E*
		L*	a*	b*	
pH 3	Control	90.15 ± 0.96^a^	2.06 ± 0.11^d^	8.81 ± 0.26^d^	—
	ZnO + PFEN0%	90.48 ± 1.19^a^	0.64 ± 0.13^e^	9.97 ± 0.35^c^	1.86 ± 0.47^d^
	ZnO + PFEN1%	63.99 ± 1.12^b^	18.93 ± 0.39^c^	15.71 ± 0.16^b^	31.88 ± 1.19^c^
	ZnO + PFEN2%	58.82 ± 1.16^c^	27.47 ± 0.15^b^	15.92 ± 0.29^ab^	40.96 ± 0.97^b^
	ZnO + PFEN3%	50.33 ± 0.89^d^	34.84 ± 0.21^a^	16.19 ± 0.24^a^	52.10 ± 1.35^a^
pH 7	Control	89.53 ± 1.10^a^	2.24 ± 0.09^a^	8.53 ± 0.42^b^	—
	ZnO + PFEN0%	89.78 ± 1.35^a^	0.62 ± 0.18^b^	10.17 ± 0.19^a^	2.32 ± 0.51^c^
	ZnO + PFEN1%	69.31 ± 1.10^b^	−0.40 ± 0.10^d^	−1.27 ± 0.31^c^	22.62 ± 0.84^b^
	ZnO + PFEN2%	68.15 ± 1.87^b^	−0.24 ± 0.13^cd^	−4.02 ± 0.19^d^	25.63 ± 1.31^a^
	ZnO + PFEN3%	68.09 ± 0.24^b^	−0.15 ± 0.08^c^	−5.39 ± 0.28^e^	25.67 ± 0.95^a^
pH 11	Control	90.26 ± 1.72^a^	2.15 ± 0.13^a^	8.60 ± 0.39^b^	—
	ZnO + PFEN0%	90.49 ± 1.44^a^	0.63 ± 0.07^b^	10.12 ± 0.33^a^	2.16 ± 0.68^d^
	ZnO + PFEN1%	62.56 ± 0.96^b^	−10.68 ± 0.19^c^	−16.18 ± 0.25^c^	39.32 ± 1.25^c^
	ZnO + PFEN2%	58.16 ± 1.02^c^	−15.11 ± 0.14^d^	−18.56 ± 0.32^d^	45.45 ± 1.12^b^
	ZnO + PFEN3%	53.39 ± 0.75^d^	−19.73 ± 0.16^e^	−22.18 ± 0.14^e^	52.78 ± 1.49^a^

*Note:* Values are mean ± SD (*n* = 3). Different superscript letters indicate significant differences (*p* < 0.05, one‐way ANOVA, *F*(4, 10) = 12.37).

Abbreviation: PFEN, pomegranate flower extract nanocapsules.

It is worth noting that this study did not include a control film containing free (non‐encapsulated) anthocyanins. While the observed improvements in film performance suggest beneficial effects of PFEN, the absence of a direct comparison with non‐encapsulated anthocyanins limits our ability to isolate the specific advantages conferred by encapsulation. Future studies should include such controls to more definitively assess the impact of encapsulation on anthocyanin stability, color responsiveness, and bioactivity within the film matrix.

### 3.4 The Relationship Between Film Color and Changes in TVB‐N Values in Fish Fillets

**FIGURE 11:**
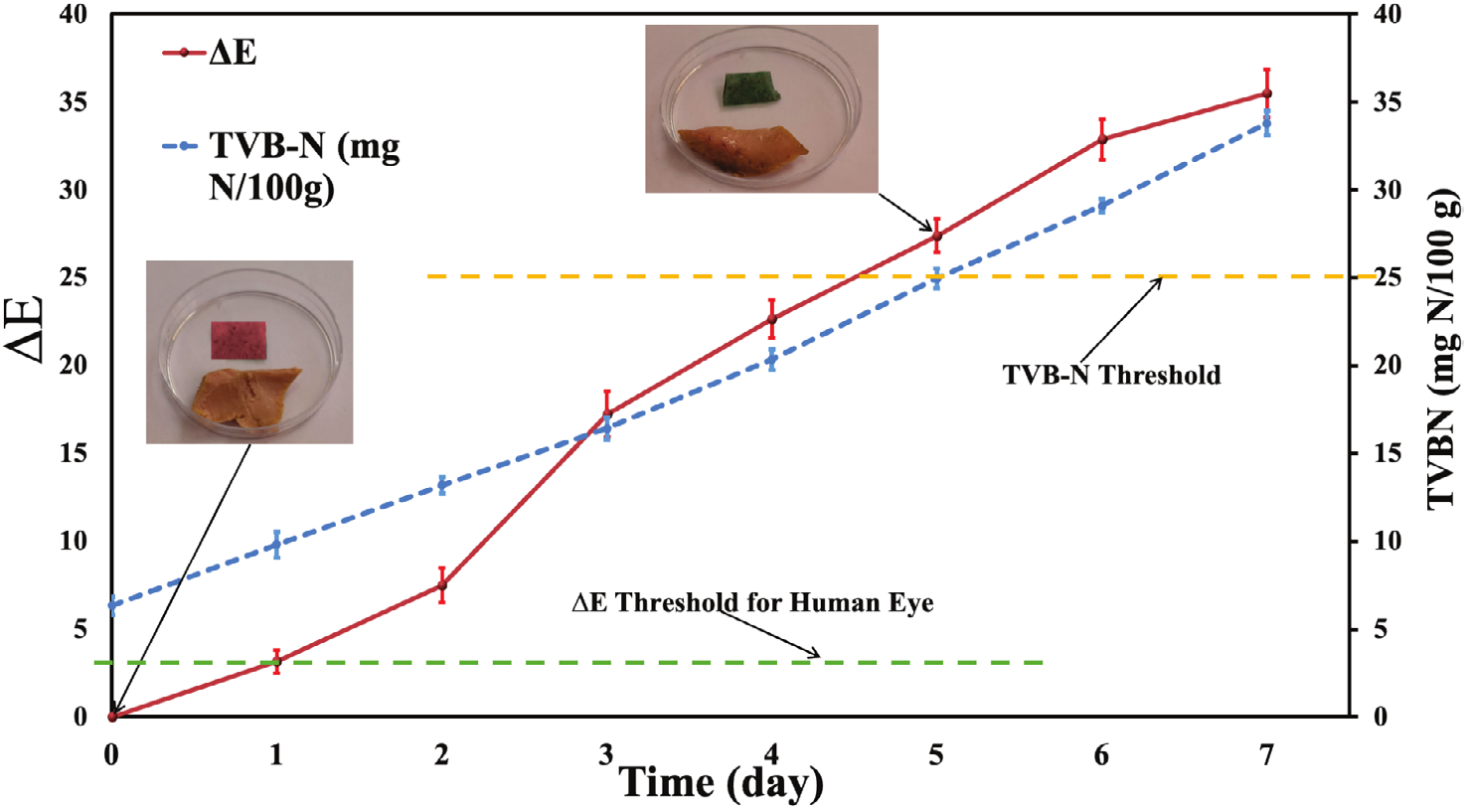
Correlation between total volatile basic nitrogen (TVB‐N, mg N/100 g) and total color difference (Δ*E*) of the smart film containing 3% PFEN, during storage of trout fillets at 4°C. Δ*E* values were calculated from color measurements of the indicator films. Error bars represent standard deviation (*n* = 3).

Fish is a meat product containing high amounts of protein, and during the storage period of these food products, as a result of the activity of microorganisms and their proteolytic enzymes, as well as internal proteases of marine products, proteins are degraded, and alkaline gases including ammonia, dimethyl, and trimethylamines are produced (Wu et al. 2021). As a result of the production of these gases, the pH of the product gradually increases over time. This study examined the color response of the chitosan/gelatin/Nano ZnO film containing 3% PFEN against alkaline gases produced during the storage period of fish fillets. Results (Figure 11) showed that at the beginning of the storage period, the TVB‐N value of the fish fillets was low due to their freshness (6.34 mg N/100 g). Over time, due to the activity of microorganisms and the production of volatile nitrogen compounds, the TVB‐N values increased significantly (*p* < 0.05). They reached 34.23 mg N/100 g on the last day of storage. Over time, the Δ*E* of the intelligent film has also changed significantly (*p* < 0.05). A significant increase in the amount of ∆E film during the storage period has been observed, indicating the color sensitivity of these films to producing volatile nitrogen compounds in the fish, and since the second day of storage, the ∆*E* values of the film were higher than 5, changing the color of the intelligent film during the storage period was recognizable by eye. The maximum recommended amount for TVB‐N in fishery products is 25 mg N/100 g (Babic Milijasevic et al. 2023).

The total color difference (Δ*E*) of the intelligent film increased from 0.78 on day 1 to 14.95 on day 7 of fish storage, indicating a progressive and quantifiable color change in response to spoilage. Notably, a Δ*E* value above 2.3 is considered the threshold for perceptible color change to the human eye. In this study, Δ*E* exceeded this threshold on day 3, corresponding to a TVB‐N value of approximately 17.6 mg N/100 g. The spoilage threshold for fish is typically considered to be 25 mg N/100 g, which was reached by day 7, when the Δ*E* reached ~15.

A linear regression was observed between Δ*E* and TVB‐N values (Δ*E* = 0.62 × TVB‐N − 2.1, *R* ^2^ = 0.94) and between Δ*E* and pH (Δ*E* = 3.45 × pH − 20.8, *R* ^2^ = 0.91). Using Δ*E* = 2.3 as the threshold for human eye detection, the limit of detection (LOD) for TVB‐N was estimated at ~10.8 mg N/100 g, which corresponds to the onset of spoilage. This indicates that the smart film can detect freshness loss before the TVB‐N level reaches the regulatory limit for fish spoilage (~25–30 mg N/100 g). Therefore, the developed film exhibits high sensitivity and practical applicability for real‐time monitoring of fish quality.

The results showed that the fish fillets had TVB‐N values below the maximum recommended level until the fifth day, and since the sixth day, their values have been higher, indicating spoilage of the fillets. Yan et al. (2021) also observed that pH and TVB‐N values in fish had a close relationship with color changes in chitosan/butterfly pudding films, and the color of the films changed from purple‐blue to dark green at the end of the storage period. You et al. (2022) also demonstrated that biopolymer films containing anthocyanin extract from sweet purple potatoes exhibited sensitivity to pH changes and ammonia levels during the shrimp storage period, resulting in clear discoloration. Zeng et al. (2023) reported that the color of the chitosan/pectin/black rice extract changed due to the spoilage of the meat, eventually reaching a blue hue.

It is well documented that anthocyanins are prone to degradation under stressors such as heat, light, and humidity, which may limit the long‐term functionality of intelligent films. Previous studies have emphasized that stability optimization (e.g., via copigmentation, protective carriers, or antioxidant co‐additives) is crucial to maintain anthocyanin responsiveness in packaging systems (Oladzadabbasabadi, Mohammadi Nafchi, Ghasemlou, et al. 2022). Although stability testing under storage conditions was not within the scope of this study, future investigations should address anthocyanin retention, color responsiveness, and mechanical/barrier property changes during prolonged storage to support real‐world applications.

## 4 Conclusion

This study successfully developed a biodegradable smart film incorporating zinc oxide (ZnO) nanoparticles and pomegranate flower extract nanocapsules (PFEN) within a chitosan/gelatin matrix for fish freshness monitoring. The composite film exhibited significantly improved functional properties, including reduced water vapor permeability (from 6.37 to 2.94 × 10^−11^ g m/m^2^ day), enhanced tensile strength (from 17.2 to 20.6 MPa), and increased antioxidant activity (up to 78.9% DPPH inhibition) (*p* < 0.05). A strong correlation was observed between the film's color change (Δ*E*) and fish spoilage levels, with Δ*E* values rising to ~15 as TVB‐N exceeded 25 mg N/100 g. These findings support the hypothesis that PFEN and ZnO synergistically enhance film performance and enable real‐time freshness detection. Future research should focus on assessing long‐term stability under varied storage conditions and directly comparing encapsulated versus free anthocyanins to further optimize the indicator's functionality.

## Author Contributions

**Solmaz Choubaki:** investigation (equal), methodology (equal), validation (equal), visualization (equal), writing – original draft (equal). **Homa Baghaei:** project administration (equal), supervision (equal), validation (equal), writing – review and editing (equal). **Abdorreza Mohammadi Nafchi:** conceptualization (equal), funding acquisition (equal), resources (equal), supervision (equal), writing – original draft (equal).

## Conflicts of Interest

The authors declare no conflicts of interest.

## Data Availability

The data that support the findings of this study are available from the corresponding author upon reasonable request.
